# Successful nonoperative management of AAST grade IV splenic injury with splenic vein thrombosis without angioembolization: a case report

**DOI:** 10.1093/jscr/rjag948

**Published:** 2026-10-22

**Authors:** Rahul Ramanujam, Padmanabhan Ragumani, Manibalan Sekar

**Affiliations:** Department of General Surgery, Chettinad Hospital and Research Institute, Chettinad Academy of Research and Education, Kelambakkam - 603103, Tamil Nadu, India; Department of General Surgery, Chettinad Hospital and Research Institute, Chettinad Academy of Research and Education, Kelambakkam - 603103, Tamil Nadu, India; Department of General Surgery, Chettinad Hospital and Research Institute, Chettinad Academy of Research and Education, Kelambakkam - 603103, Tamil Nadu, India

**Keywords:** splenic injury, blunt abdominal trauma, nonoperative management, splenic vein thrombosis, angioembolization, case report

## Abstract

High-grade splenic trauma remains challenging, particularly when associated with vascular complications. We report a man in his 20s who sustained blunt abdominal trauma and was diagnosed on contrast-enhanced computed tomography with an American Association for the Surgery of Trauma grade IV splenic injury, moderate haemoperitoneum, and splenic vein thrombosis without active arterial contrast extravasation. Although initially hypotensive, he stabilized after resuscitation with three units of packed red blood cells and three units of fresh frozen plasma. He was successfully managed nonoperatively with close clinical monitoring, serial haemoglobin assessment, and interval imaging. Follow-up imaging demonstrated reduction of haemoperitoneum and partial recanalization of the splenic vein, with complete radiological resolution at 10 months. This case demonstrates that carefully selected patients with high-grade splenic injury and traumatic splenic vein thrombosis may be managed successfully without angioembolization when haemodynamic stability is achieved and maintained.

## Introduction

Splenic injury is among the most frequent consequences of blunt abdominal trauma, particularly after road traffic accidents. Advances in trauma care have shifted management from routine splenectomy to selective splenic preservation in haemodynamically stable patients. However, high-grade injuries remain challenging owing to the increased risk of delayed haemorrhage and failure of conservative treatment.

Traumatic splenic vein thrombosis following blunt splenic injury is rarely reported, and its influence on management remains uncertain. We present a case of successful nonoperative management of an AAST grade IV splenic injury complicated by splenic vein thrombosis without angioembolization [1–3].

## Case report

A man in his twenties presented to the emergency department of our tertiary care centre one day after a road traffic accident with diffuse abdominal pain and distension. On arrival, he was hypotensive (80/50 mmHg) and tachycardic (122 bpm), with a Glasgow Coma Scale score of 15. Focused sonography for trauma was positive for free intraperitoneal fluid. Contrast-enhanced computed tomography (CT) of the abdomen demonstrated splenic enlargement with extensive lacerations involving the hilar region, moderate haemoperitoneum of ~600 ml, and a 1.5 cm thrombus in the splenic vein at the hilar level, consistent with AAST grade IV splenic injury with associated splenic vein thrombosis (Figs 1 and 2). No active arterial contrast extravasation or pseudoaneurysm was identified.

**Figure 1:**
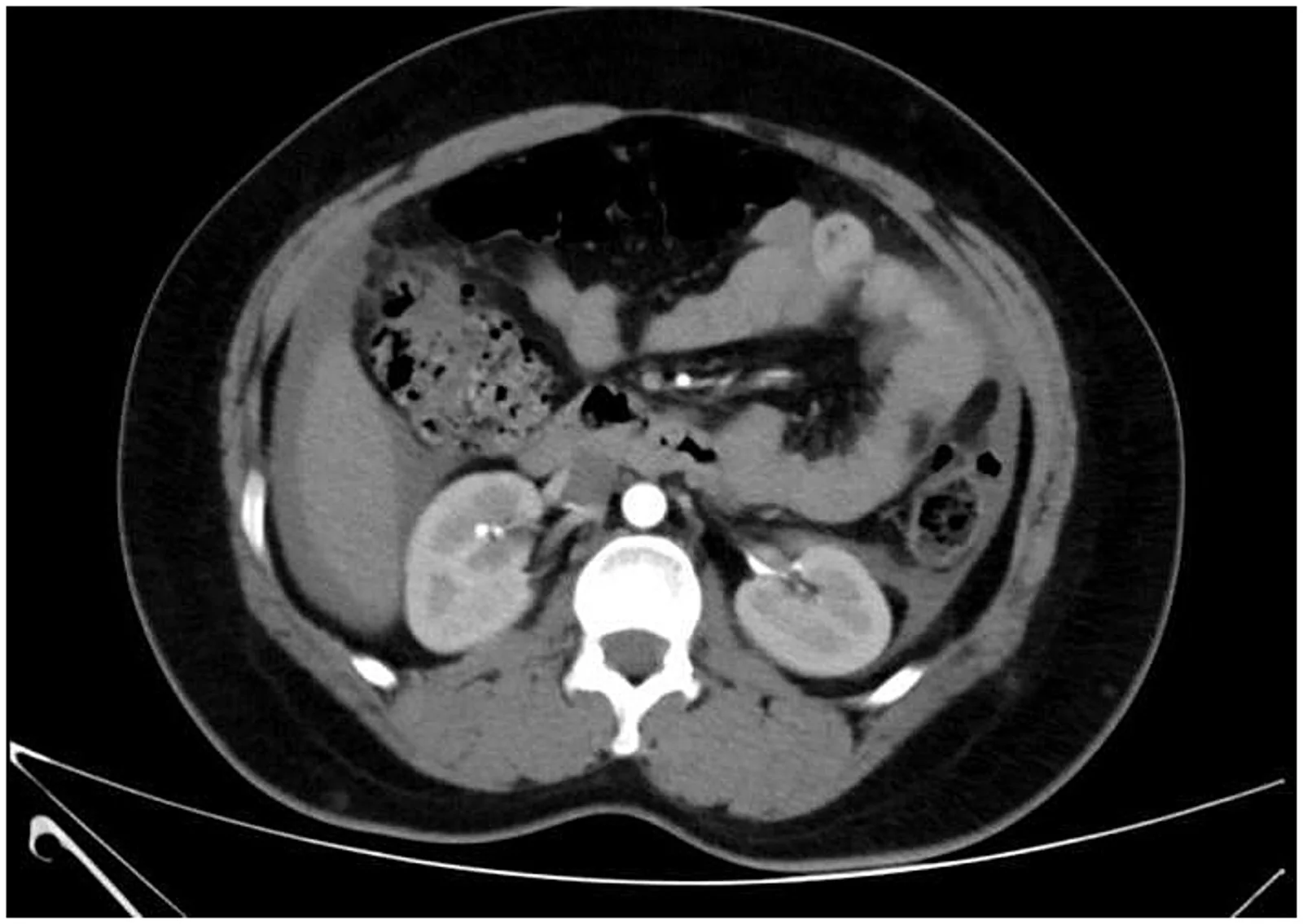
Initial contrast-enhanced CT demonstrating splenic laceration involving the hilar region with associated haemoperitoneum.

**Figure 2:**
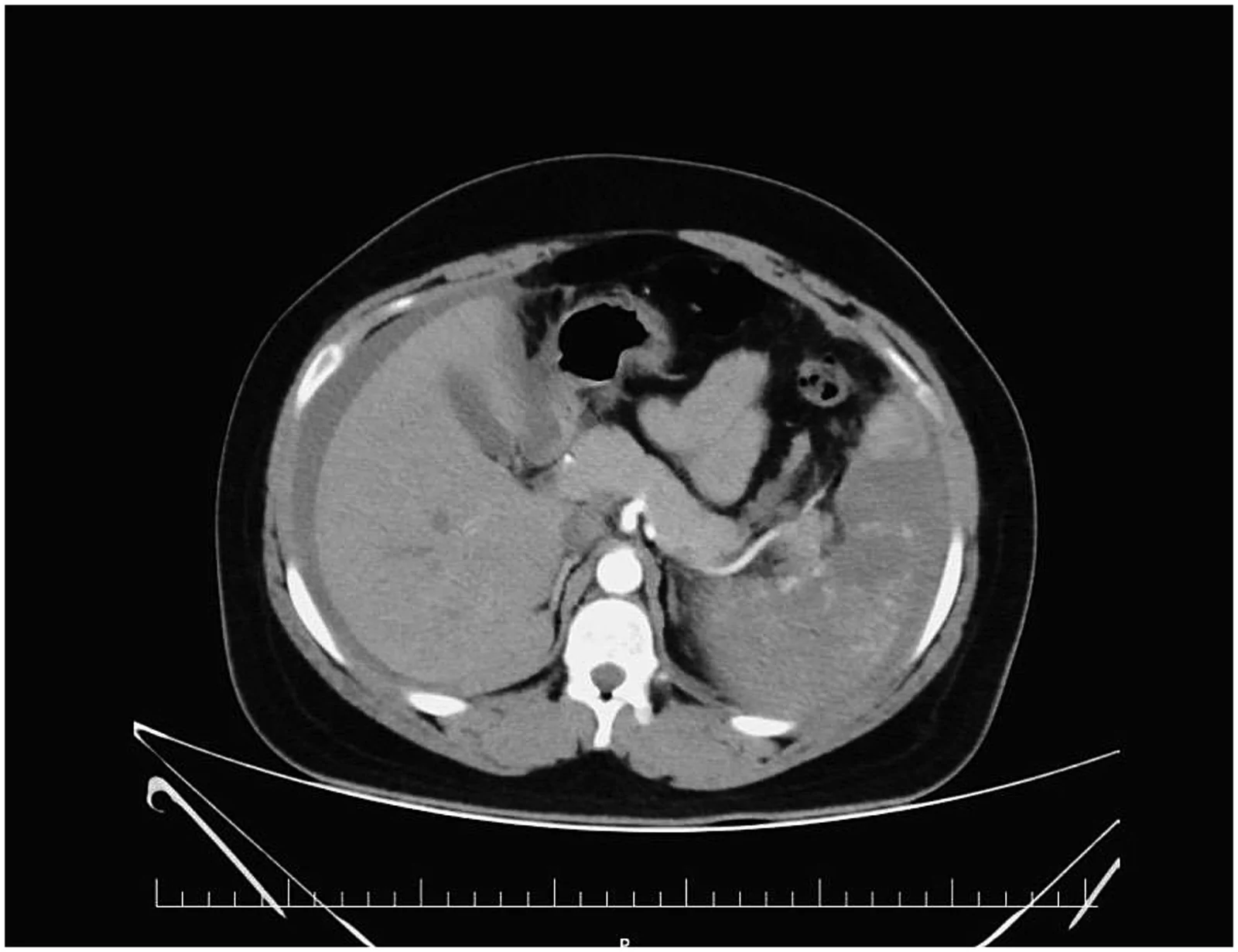
Initial CT demonstrating splenic vein thrombosis at the hilar level without active contrast extravasation.

Initial haemoglobin was 4.9 g/dL with thrombocytopenia. The patient was admitted to the intensive care unit and underwent prompt resuscitation with intravenous fluids, 3 units of packed red blood cells and 3 units of fresh frozen plasma. Haemodynamic stability was achieved after resuscitation and sustained. As serial examinations showed no further deterioration, nonoperative management was adopted after multidisciplinary review, with angioembolization and laparotomy reserved for worsening.

Observation was structured and intensive, with close monitoring of vital signs, abdominal girth, and haemoglobin levels. Supportive care included analgesia, antibiotic cover, and venous thromboembolism prophylaxis according to clinical status. Anticoagulation was not initiated during the acute phase due to haemorrhagic risk, with radiological monitoring of the thrombus. Interval CT performed during admission demonstrated progressive reduction in the volume of haemoperitoneum, no new hematoma or pseudoaneurysm, and partial recanalization of the splenic vein (Figs 3 and 4). Haemoglobin improved steadily to 12.8 g/dL over 9 days without further transfusion after initial stabilization.

**Figure 3:**
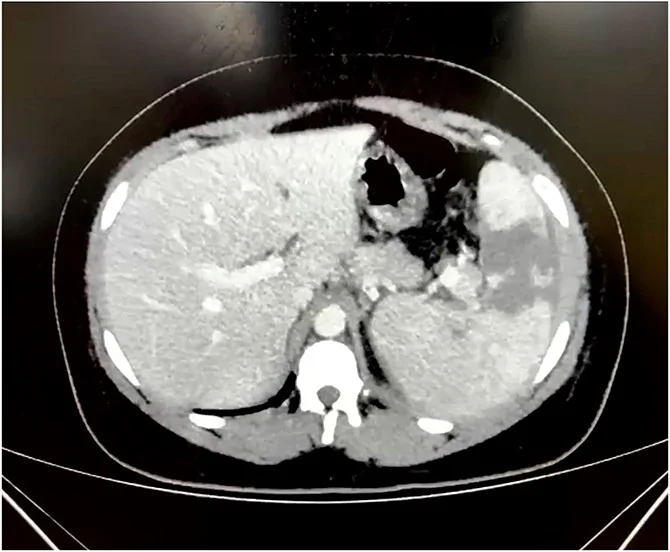
Follow-up CT demonstrating interval reduction in hemoperitoneum with partial recanalization of the splenic vein during nonoperative management in the hospital.

**Figure 4:**
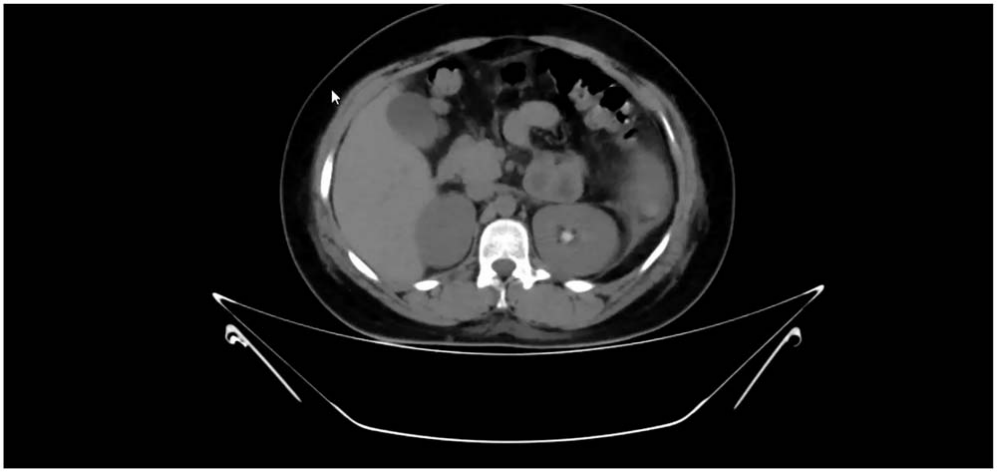
Follow-up CT demonstrating progressive interval reduction in hemoperitoneum during nonoperative management in the hospital.

The patient tolerated oral intake by Day 9 was transferred from the intensive care unit to the ward after continued stability and was discharged on Day 14. At 30-day review, he had resumed routine daily activity, and repeat imaging showed only minimal residual haemoperitoneum without new complications. Continued follow-up over the subsequent 10 months demonstrated complete radiological resolution.

## Discussion

This case highlights the importance of integrating physiological response with anatomical injury grading when managing splenic trauma. Although AAST grade IV injuries are associated with an increased risk of nonoperative management (NOM) failure, injury grade alone should not dictate operative intervention. Current Eastern Association for the Surgery of Trauma and World Society of Emergency Surgery guidelines recommend NOM for appropriately selected patients who achieve sustained haemodynamic stability [1, 2].

The coexistence of high-grade splenic injury and traumatic splenic vein thrombosis is uncommon, and evidence guiding management remains limited. Most published literature on vascular complications following blunt splenic trauma focuses on arterial lesions, including contrast blush, pseudoaneurysm, and arteriovenous fistula, which are recognized predictors of ongoing haemorrhage and established indications for angiography with embolization. In contrast, the clinical significance of traumatic splenic vein thrombosis is poorly defined, with few reports describing its natural history or optimal management.

Despite initial hypotension, our patient demonstrated a sustained response to blood product resuscitation and had no CT evidence of active arterial bleeding or pseudoaneurysm. Serial clinical assessment, stable haemoglobin following initial transfusion, and interval CT showing progressive reduction of haemoperitoneum with partial splenic vein recanalization supported continuation of NOM. Anticoagulation was deferred during the acute phase because the immediate risk of haemorrhage outweighed the potential benefit of thrombus treatment and no thrombotic or haemorrhagic complications developed during follow-up.

Although prophylactic angioembolization has been advocated for selected high-grade splenic injuries, its routine use remains controversial in the absence of arterial vascular injury. In this patient, the absence of radiological features warranting endovascular intervention and continued haemodynamic stability allowed safe observation without embolization. This case supports an individualized approach in which angiography is reserved for patients with active arterial bleeding or clinical deterioration rather than for injury grade alone [3, 4].

Successful NOM of complex splenic trauma requires careful patient selection, multidisciplinary decision-making, and close clinical surveillance, access to blood products and repeat imaging, and readiness to intervene if deterioration occurs. This case adds to the limited evidence that traumatic splenic vein thrombosis may not necessarily preclude splenic preservation and that, in selected patients without arterial vascular injury, successful management without angioembolization is achievable [1–6].

## Timeline table

The clinical course from Day 0 to 10 months is summarized in Table 1, including presentation after road traffic accident, initial haemodynamic instability, CT findings of AAST grade IV splenic injury with splenic vein thrombosis, ICU resuscitation with blood products, nonoperative management, serial imaging improvement, discharge on Day 14, return to routine activity by Day 30, and complete radiological resolution at 10 months.

**Table 1:** Table summarizing the clinical course from Day 0 to 10 months, including presentation after road traffic accident, initial haemodynamic instability, CT findings of AAST grade IV splenic injury with splenic vein thrombosis, Intensive Care Unit resuscitation with blood products, NOM, serial imaging improvement, discharge on Day 14, return to routine activity by Day 30, and complete radiological resolution at 10 months

**Clinical timeline**	**Clinical presentation, investigations, and management**
Day 0	Sustained road traffic accident
Day 1	Presented to casualty with diffuse abdominal pain and abdominal distension with initial haemodynamic instability. Positive focused assessment with sonography for Trauma scan Contrast-enhanced CT abdomen: moderate haemoperitoneum (600 ml) and AAST Grade IV splenic injury with splenic vein thrombosis at the splenic hilum level with a length of 1.5 cm, indicative of splenic vein injury. Hb on presentation: 4.9 Cautious serial second-hourly haemoglobin and vital signs
Day 2	1 unit packed red blood cells and 1 unit fresh frozen plasma were transfused under adequate diuretic cover Haemodynamically stable post-transfusion. Taken up for NOM under the backdrop of the potential need for angioembolization and surgery in case of clinical deterioration. Prophylactic venous thromboembolic therapy initiated.
Day 3–4	Interval CT abdomen scan on Day 4: decreasing haemoperitoneum (~500 ml), associated with possible splenic infarcts in addition to the previous findings - No further clinical deterioration noted with an upward trend in the hemoglobin levels
Day 5–8	Interval contrast-enhanced CT abdomen scan on Day 8: decreasing haemoperitoneum (~400 ml) with partial recanalization of splenic vein - Amidst clinical and radiological improvement, orals were initiated and tolerated.
Day 9	Mobilized. Hemodynamically stable throughout ICU follow-up stay with no subsequent need for transfusion. Abdominal girth decreased. Abdominal pain relieved significantly. Shifted from intensive care unit to ward
Day 10–13	Interval CT abdomen: decreasing haemoperitoneum (~200 ml) Hemodynamically stable throughout the hospital stay. Other injuries managed conservatively.
Day 14	Discharged with follow-up advice
Day 30	Resumed routine activities on Day 30 CT abdomen on Day 30: minimal hemoperitoneum (<100 ml)
Follow up	Complete resolution of the haemoperitoneum at the 10-month follow-up period was achieved.

## Highlights

1. Rare combination of AAST grade IV splenic injury with traumatic splenic vein thrombosis.
2. Management required balancing the competing risks of haemorrhage and thrombosis.
3. Haemodynamic stability after resuscitation allowed splenic preservation without arterial intervention.
4. Serial CT demonstrated spontaneous venous recanalization without endovascular intervention.
5. This case expands the limited evidence guiding management of traumatic splenic vein thrombosis.
6. Supports individualized management beyond injury grade in selected high-grade splenic trauma.
